# Sociodemographic profiles and organ damage accural in the Black Women’s Experience Living with Lupus study

**DOI:** 10.1177/09612033231218923

**Published:** 2023-12-04

**Authors:** Connor D Martz, Tamika Webb-Detiege, Maria I Danila, David H Chae

**Affiliations:** 1 Population Research Center, 12330 The University of Texas at Austin, Austin, TX, USA; 2 Department of Social, Behavioral, and Population Sciences, 25812 Tulane University School of Public Health and Tropical Medicine, New Orleans, LA, USA; 3 Department of Rheumatology, 633467 Ochsner Health, New Orleans, LA, USA; 4 The University of Queensland Medical School, 589787 Ochsner Clinical School, New Orleans, LA, USA; 5 Division of Clinical Immunology and Rheumatology, Department of Medicine, 164494 The University of Alabama at Birmingham, Birmingham, AL, USA; 6 Geriatric Research Education and Clinical Center, 19957 Birmingham VA Medical Center, Birmingham, AL, USA

**Keywords:** socioeconomic, systemic lupus erythematosus, racial disparities, organ damage

## Abstract

**Objective:**

Black/African American women with systemic lupus erythematosus (SLE) experience greater organ damage and at younger ages than white women. The objective of this study was to advance research on SLE inequities by identifying sociodemographic risk profiles associated with organ damage accrual specifically among Black/African American women.

**Methods:**

Latent profile analysis was conducted among 438 Black/African American women with SLE living in Atlanta, GA and enrolled in the Black Women’s Experiences Living with Lupus (BeWELL) Study (May 2015 to April 2017). Proportional hazard and Poisson regression models examined prospective associations between sociodemographic profiles and the timing and degree of organ damage accrual over 2 years.

**Results:**

Four profiles emerged: (1) “Younger/Lower SES with Uncontrolled SLE” (44.8%), (2) “Older/Lower SES with Uncontrolled SLE” (23.3%), (3) “Mid-SES with Controlled SLE” (19.6%), and (4) “Higher SES with Controlled SLE” (11.2%). Approximately 42% of participants experienced new organ damage during the follow-up period. Proportional hazard models indicated that “Older/Lower SES with Uncontrolled SLE” participants were at greatest risk of new organ damage (HR = 2.41; 95% CI = 1.39, 4.19), followed by “Younger/Lower SES with Uncontrolled SLE” participants (HR = 1.56; 95% CI = 0.92, 2.67), compared to those in the “Higher SES with Controlled SLE” profile. Poisson regression models revealed that these two groups also exhibited greater organ damage accrual (b = 0.98, SE = 0.24, 95% CI = 0.52, 1.44 and b = 0.72, SE = 0.23, 95% CI = 0.27, 1.17, respectively).

**Conclusions:**

Black/African American women with fewer socioeconomic resources and uncontrolled SLE are at greatest risk for increasing disease severity over time. Social inequities likely contribute to racial inequities in SLE progression.

## Introduction

Black/African Americans in the U.S. have higher prevalence of systemic lupus erythematosus (SLE), experience faster disease progression, greater disease severity, and worse SLE outcomes, including increased mortality that also occurs at earlier ages, compared to their white counterparts.^1,2^ Although genetic risk factors for SLE exist, their role in explaining racial inequities in SLE is limited.^3,4^

SLE activity is driven by inflammatory mechanisms and, when uncontrolled, results in irreversible organ damage, such as kidney failure, cardiovascular disease, and pulmonary disorders.^2,3,5^ Organ damage accrual and the rate at which it occurs are important SLE outcomes that predict disability, reduced quality of life, and mortality.^3,6,7^ For example, organ damage accrual within a year of diagnosis has been linked to a 3–4 fold increased risk of mortality, and existing damage increases risk of new damage accrual.^8,9^ Several studies have reported that Black/African Americans with SLE are at greater risk for organ damage accrual and mortality.^10,11^ Black/African Americans also experience higher rates of initial damage (i.e., transition from none to at least some) and damage accumulation (i.e., transition from some damage to greater damage), compared to white SLE patients.^12^

Emerging research suggests the causes of racial inequities in SLE progression and outcomes are rooted in social inequities rather than biological differences in disease susceptibility.^1,4,13^ For example, SLE disease activity is sensitive to psychosocial stressors unique to the lived experience of Black/African Americans, such as racial discrimination and other forms of racism-related stress.^14–17^ Other research has found that Black/African American women with SLE living in racially segregated neighborhoods face increased risk of depression—a common comorbidity associated with organ damage in this population.^18,19^ Structural racism also undermines socioeconomic mobility for Black/African Americans, which in turn reduces access to health promoting resources and increases exposure to risk factors for SLE disease progression, including organ damage accrual.^20–23^

Most research on correlates of organ damage accrual has focused on clinical (e.g., disease activity, medication use) or basic demographic (e.g., age, gender, race/ethnicity) factors. A limited number of studies have identified poverty and socioeconomic disadvantage as risk factors for damage accrual.^10,20,24^ However, much of this work has been conducted in predominantly white or racially diverse samples with low proportions of Black/African American women. These limitations mask within-racial group variability and preclude the identification of sociodemographic risk factors specifically among Black/African American women, a population that experiences disproportionate SLE burden. Moreover, most studies have relied on convenience or otherwise self-selected samples that may have different sociodemographic characteristics from the underlying population of interest. When attempting to identify subgroup patterns within a population, person-centered approaches (i.e., latent profile analysis) offer advantages over traditional variable-centered procedures (i.e., regression) by considering the intersection between multiple disease and sociodemographic characteristics and how they may cluster together. Profiles that emerge from a latent profile analysis (LPA) may be useful in clinical settings to identify patients at greatest risk of organ damage.

To our knowledge, there are no prospective studies which identify high-risk sociodemographic profiles of organ damage accural specifically among Black/African American women, despite this group being at greatest risk for accelerated disease progression. In the current study, we identified sociodemographic, socioeconomic, and health-related profiles of Black/African American women with SLE and examined prospective associations with organ damage accrual over a 2-year period.

## Methods

### Sample

The Black Women’s Experiences Living with Lupus (BeWELL) Study enrolled 438 participants living in metropolitan Atlanta, GA, from May 2015 to April 2017.^14^ All participants had validated SLE based on criteria set by the American College of Rheumatology, and were recruited largely from a population-based registry with supplemental sampling via regional clinics, labs, and providers. The BeWELL sample is characterized by varying levels of disease severity and socioeconomic resources. There were three main waves of data collection (baseline, 1-year follow-up, and 2-year follow-up). Data were collected between May 2015 and February 2019. Participant engagement was high, with more than 95% of survivors (n deceased = 19) completing all three waves.

### Variables

Baseline measures of demographic, socioeconomic, and health characteristics were used in the latent profile analysis. Continuous measures were age in years, calculated using participant date of birth; disease duration in years, based on self-reported month and year when they were diagnosed, how long ago they were diagnosed, or their age at diagnosis (whichever was volunteered); and income-to-poverty ratio, measured continuously and calculated as self-reported annual household income before taxes divided by the poverty threshold (based on household size and number of children).^25^ Categorical variables included relationship status (married or marriage-like; romantic relationship; divorced, widowed, or separated; and single), work status (full-time, part-time, out of labor force, and unable to work due to health or disability), insurance status (private, public, uninsured), and educational attainment (less than high school, high school graduate or equivalent, some college, and college degree).

SLE-related measures included disease activity and organ damage. Disease activity was measured continuously using the Systemic Lupus Activity Questionnaire (SLAQ), a validated self-report instrument designed for epidemiological research that assesses SLE symptoms in the past 3 months, such as skin rashes, fatigue, fever, and joint swelling. Possible scores range from 0 (no disease activity) to 44 (high disease activity). The Brief Index of Lupus Damage (BILD) assessed damage across 12 organ systems due to SLE.^26,27^ The BILD is a validated, self-report tool used in clinical and epidemiological SLE research, with greater damage scores shown to predict quality of life, disability, and mortality.^26,27^ Items assessed cumulative and irreversible damage to an organ or organ system since the onset of SLE which has been present for at least 6 months. Possible scores range from 0 to 30, with higher scores indicating greater organ damage. Self-reported SLE medication use at baseline was assessed in-person through an interviewer-administered questionnaire.^14^ Prior to their interview, participants were reminded to bring information on their current SLE medication use. Trained interviewers also went through a checklist of SLE medications with each participant. In the current study, SLE medication use was scored dichotomously (yes vs no) for the following: glucocorticoids (e.g., prednisone, medrol, methylprednisolone), hydroxychloroquine, and other immunosuppressants (e.g., methotrexate, cyclophosphamide, cyclosporine, mycophenolate, dapsone, azathioprine, belimumab, rituximab).

We examined time to first accrual of new organ damage and cumulative damage accrual over two annual assessments. New organ damage was assessed as an increase in BILD score since baseline with death considered new organ damage. Time to new organ damage was calculated as the number of years between the date of baseline assessment and the date of assessment when new organ damage was identified. For participants that died before their first annual assessment, time to event was coded as 1 year. For participants that died between their first and second annual assessment, time to event was calculated as the time between baseline and the first annual assessment, plus 1 year. The BeWELL Study tracked mortality through the duration of data collection, including for participants who had already completed their final assessment. Accordingly, time to event for participants that did not experience new organ damage across two annual assessments but died after their final assessment was calculated as the number of years between baseline and their date of death.

A count variable for cumulative organ damage was calculated as the difference in BILD scores between baseline and the final completed assessment. No damage accrual or death was scored as 0; a one unit increase in BILD was scored as 1; a 2 unit increase in BILD was scored as 2; an increase in BILD of 3 or more was scored as 3 due to few participants with an increase in BILD of 4 or more units. Death was scored as 4.

### Analytic plan

We used latent profile analysis (LPA) to identify subgroups or profiles of participants in the BeWELL Study based on sociodemographic, socioeconomic, and healthcharacteristics. LPA is a exploratory modeling approach to group participants who have similar characteristics across various sociodemographic, clinical, and treatment-related domains.^28,29^ It is useful for identifying complex patterns in observed characteristics that represent nuanced latent constructs or hidden clinical phenotypes.^29^ The probability of each participant belonging to a specific subgroup is calculated using maximum likelihood estimation, and participants are assigned to their most likely subgroup (i.e., highest probability of belonging).^28^ If model entropy is high (>0.8) and suggests clear distinction between subgroups, the resulting profiles can then be used as a predictor or outcome variable in an auxiliary model.

The best-fitting solution in LPA was determined by empirical fit indices as well as model parsimony.^30^ Models of increasing profile size were used to compare fit indices of BIC and aBIC (lower is better), entropy (values closer to 1 indicate good fit), and the bootstrapped likelihood ratio test (BLRT, which statistically compares the fit of *k* vs *k*-1 profile solutions (*p* < .05 indicates a significantly better fit to the data).^28^ We also examined relative gains in model fit through elbow plots of BIC values, and considered theoretical and conceptual interpretations of identified profiles.^28^ After identifying a best-fitting solution, posterior probabilities and the most likely profile assignment were used for survival analyses. ANOVAs and chi-square tests identified whether LPA indicators significantly differed across profiles. Missing data for sociodemographic, socioeconomic, and SLE-related indicator variables was minimal (<1%) and accounted for using full-information maximum likelihood.

Next, we examined whether sociodemographic profiles were associated with the time to first new organ damage and cumulative organ damage accrual. Non-deceased participants lost to follow-up after baseline (n = 11) were excluded from analyses resulting in a final analytic sample size of 427. Time to event data was interval-censored for participants who experienced new organ damage because the exact timing of damage accrual was unknown. For each participant *i* that experienced new organ damage, a response interval (*L*_*i*_, *R*_*i*_) was calculated, where *L*_*i*_ is the last follow-up time at which the event had not occurred, and *R*_*i*_ is the last follow-up time immediately after the event. Participants were right censored if they did not experience damage accrual or death. Survival functions were estimated for each profile and comparisons were made using generalized log-rank statistics. Proportional hazard regression models were then fit to examine the hazard function of each profile.

We also examined associations between sociodemographic profiles and the amount of new organ damage over 2 years using Poisson regression. Organ damage accumulation was modeled continuously using the following categories: no increase; BILD score increases of 1, 2 and 3 or more; and death. Estimates were calculated in referent to Higher SES with Controlled SLE participants. LPA was performed in Mplus and survival analyses were performed in SAS 9.4.

## Results

On average, participants were 46.84 years old (SD = 12.31) at baseline and had been living with SLE for 15.95 years (SD = 10.37). Approximately 42% of the sample experienced damage to a new organ or organ system over the follow-up period (M = 0.68, SD = 0.99, range 0–5).

### Latent profile analysis

Results of the LPA indicated a 4-profile solution best fit the data (Table S1). Profile sociodemographic characteristics are presented in Table 1. The first profile was characterized by indicators of low socioeconomic standing, moderate educational attainment, and moderate SLE severity with uncontrolled disease activity. Relative to other profiles, participants assigned to Profile 1 were younger (M = 39.61, SD = 9.64) with a shorter disease duration (M = 10.62 years, SD = 6.34), and had the lowest income-to-poverty ratio (M = 1.07, SD = 0.61). Most participants in Profile 1 reported being unable to work due to SLE (69.90%), not having a college degree (86.22%), and having public insurance (70.92%). These participants also had high disease activity (M = 17.22, SD = 7.92), moderate levels of organ damage (M = 2.41, SD = 2.50), and high rates of glucocorticoid (72.96%) and hydroxychloroquine (81.63%) use. Profile 1 represents the largest of the sample (44.75%) and was named “Younger/Lower SES with Uncontrolled SLE.”

**Table 1.** Sample characteristics by sociodemographic profiles in the BeWELL study (*n* = 438).

	BeWELL sample (*n* = 438)	Younger/Lower SES with uncontrolled SLE (*n* = 196)	Older/Lower SES with uncontrolled SLE (*n* = 107)	Mid-SES with controlled SLE (*n* = 86)	Higher SES with controlled SLE (*n* = 49)		
Variable	M (SD) or n (%)	M (SD) or n (%)	M (SD) or n (%)	M (SD) or n (%)	M (SD) or n (%)	F or χ2	Tukey
Age	46.84 (12.31)	39.61 (9.64)	59.07 (7.62)	45.50 (10.90)	51.38 (9.64)	102.12^***^	2>4>3>1
Years since diagnosis	15.95 (10.37)	10.62 (6.34)	26.10 (9.86)	14.74 (9.12)	17.22 (10.07)	80.52^***^	2>4>1; 2>3>1
Disease activity	15.11 (7.97)	17.22 (7.92)	17.14 (7.33)	10.65 (6.55)	10.04 (6.26)	26.42^***^	1>3; 1>4; 2>3; 2>4
Organ damage	2.55 (2.52)	2.41 (2.50)	3.75 (2.49)	1.49 (1.61)	2.33 (2.95)	14.79^***^	2>4; 2>1>3
Relationship status (*n*, %)						46.10^***^	
Married/Marriage-like	198 (45.21)	83 (42.35)	48 (44.86)	37 (43.02)	30 (61.22)	1.97	–
Romantic relationship	27 (6.16)	15 (7.65)	2 (1.87)	8 (9.30)	2 (4.08)	2.01	–
Divorced/Widowed/Separated	96 (21.69)	28 (14.29)	43 (40.19)	17 (19.77)	7 (14.29)	10.50^***^	2>3; 2>4; 2>1
Single	118 (26.94)	70 (35.71)	14 (13.08)	24 (27.91)	10 (20.41)	6.63^***^	1>2
Income-poverty ratio	2.00 (1.68)	1.07 (0.61)	1.55 (0.76)	2.48 (0.96)	5.91 (1.14)	496.96^***^	4>3>2>1
Work status (*n*, %)						254.30^***^	
Full time	125 (28.54)	25 (12.76)	1 (0.93)	67 (77.91)	32 (65.31)	120.65^***^	3>1>2; 4>1>2
Half time	54 (12.33)	32 (16.33)	7 (6.54)	13 (15.12)	2 (4.08)	3.35^*^	–
Out of labor force	22 (5.02)	2 (1.02)	11 (10.28)	1 (1.16)	8 (16.33)	10.10^***^	2>1; 2>3; 4>1; 4>3
Unable to work	237 (54.11)	137 (69.90)	88 (82.24)	5 (5.81)	7 (14.29)	88.17^***^	2>1>3; 2>1>4
Insurance (*n*, %)						247.13^***^	
Private	157 (35.84)	20 (10.20)	18 (16.82)	81 (94.19)	28 (77.55)	170.93^***^	3>4>1; 3>4>2
Public	233 (53.20)	139 (70.92)	82 (76.64)	4 (4.65)	8 (16.33)	80.42^***^	1>3; 1>4; 2>3; 2>4
None	48 (10.96)	37 (18.88)	7 (6.54)	1 (1.16)	3 (6.12)	8.52^***^	1>2; 1>3; 1>4
Education (*n*, %)						98.10^***^	
< High School	37 (8.47)	17 (8.67)	17 (15.89)	1 (1.18)	2 (4.08)	5.05^**^	2>3
High school	79 (18.08)	51 (26.02)	20 (18.69)	5 (5.88)	3 (6.12)	7.61^***^	1>4; 1>3
Some college	197 (45.08)	101 (51.53)	51 (47.66)	33 (34.12)	16 (32.65)	3.77^*^	1>3
College degree	124 (28.38)	27 (13.78)	19 (17.76)	51 (58.82)	28 (57.14)	34.35^***^	4>1; 4>2; 3>1; 3>2
Glucocorticoid use (*n*, %)	224 (55.71)	143 (72.96)	47 (43.93)	35 (40.70)	19 (38.78)	43.21^***^	1>2; 1>3; 1>4
Hydroxychloroquine use (*n*, %)	319 (72.83)	160 (81.63)	58 (54.21)	68 (79.07)	33 (67.35)	28.87^***^	1>2; 3>2
Other immunosuppressant use (*n*, %)	195 (44.52)	110 (56.12)	29 (27.10)	36 (41.86)	20 (40.82)	24.34^***^	1>2

Note: ^*^p < .05, ^**^p < .01, ^***^p < .001. Profile numbers presented for Tukey tests correspond to 1 = “Younger/Lower SES with Uncontrolled SLE” and so forth, from left to right.

The second profile had similar low socioeconomic characteristics as Profile 1, but participants in Profile 2 were older (M = 59.07, SD = 7.62) and had been living with SLE the longest (M = 26.10, SD = 9.86). Participants assigned to Profile 2 had the most severe SLE, with high disease activity (M = 17.14, SD = 7.33) and the highest levels of organ damage (M = 3.75, SD = 2.49) at baseline compared to other profiles. Profile 2 had a higher income-to-poverty ratio compared to Profile 1, but is still characterized as living near poverty (M = 1.55, SD = 0.76). Most participants in Profile 2 were unable to work (82.24%), had public insurance (76.64%), and did not have a college degree (82.24%). These participants also reported lower use of glucocorticoids, hydroxychloroquine, and immunosuppressants compared to other profiles. About 23.29% of the sample (*n* = 107) is represented in Profile 2, which we named “Older/Lower SES with Uncontrolled SLE.”

Profile 3 is characterized as working-class with low disease severity and moderate socioeconomic resources. Most participants in Profile 3 were working (93.03%), either full-time (77.91%) or part-time (15.12%), and had a significantly higher income-to-poverty ratio (M = 2.48, SD = 0.96) than the first two profiles. Black/African American women assigned to Profile 3 were educated (92.24% reported at least some college with 58.82% having obtained a college degree) and most likely to have private insurance (94.19%). Baseline levels of organ damage were lowest for Profile 3 (M = 1.49, SD = 1.61), which also had lower levels of disease activity (M = 10.65, SD = 6.55) than Profiles 1 and 2, and high use of hydroxychloroquine (79.07%). About 19.63% of the sample was assigned to Profile 3, which we named “Mid-SES with Controlled SLE.”

The final profile is distinguished by high levels of socioeconomic resources, with the highest income-to-poverty ratio (M = 5.91, SD = 1.14) and educational attainment (57.14% had a college degree). Most participants in this profile worked full-time (65.31%) and had private insurance (77.55%). Profile 4 is also described by moderate levels of SLE severity at baseline, with lower disease activity (M = 10.04, SD = 626) then Profiles 1 and 2, and less organ damage (M = 2.33, SD = 2.95) than Profile 2. This final profile represents the smallest of the sample (11.19%) and was named “Higher SES with Controlled SLE.”

### Survival analyses

Results of generalized log-rank tests indicate that overall survival rates differed across all four profiles (χ2 = 18.83, *p* < .0001). Results of proportional hazards regression models fit to the interval-censored data are presented in Table 2. Participants in the “Older/Lower SES with Uncontrolled SLE” profile had greater risk of organ damage accrual compared to the “Higher SES with Controlled SLE” profile (HR = 2.41; 95% CI = 1.39, 4.19). “Younger/Lower SES with Uncontrolled SLE” participants were also at increased risk of new organ damage, although this estimate did not reach statistical significance (HR = 1.56; 95% CI = 0.92, 2.67). Participants in the “Mid-SES with Controlled SLE” profile experienced similar risk of organ damage accrual as the “Higher SES with Controlled SLE” profile (HR = 1.01; 95% CI = 0.54, 1.88).

**Table 2.** Results of proportional hazard and Poisson regression models examining associations between profile membership and time to first damage accrual and cumulative damage accrual in the BeWELL study (*n* = 427).

Proportional hazard models		
Profile comparison	Hazard ratio	95% CI
Younger/Lower SES with Uncontrolled SLE vs Higher SES with Controlled SLE	1.56	(0.92, 2.67)
Older/Lower SES with Uncontrolled SLE vs Higher SES with Controlled SLE	2.41	(1.39, 4.19)
Mid-SES with Controlled SLE vs Higher SES with Controlled SLE	1.01	(0.54, 1.88)

Poisson regression models		
Profile	b (SE)	95% CI
Younger/Lower SES with Uncontrolled SLE	0.72 (0.23)	(0.27, 1.17)
Older/Lower SES with Uncontrolled SLE	0.98 (0.24)	(0.52, 1.44)
Mid-SES with Controlled SLE	0.10 (0.27)	(−0.43, 0.62)
Higher SES with Controlled SLE	(ref)	(ref)

Note: proportional hazard models examined the time to first new organ damage accrual. Poisson regression models examined the amount of new organ damage accrual over the 2-year study period.

Poisson regression models examined associations between profiles and the amount of organ damage accrual, measured continuously in the following categories: no increase; BILD score increases of 1, 2 and 3 or more; and death (Table 2). Results indicate that compared to “Higher SES with Controlled SLE” participants, greater organ damage accrual was most strongly associated with participants in the “Older/Lower SES with Uncontrolled SLE” profile (b = 0.98, SE = 0.24, 95% CI = 0.52, 1.43), followed by those in the “Younger/Lower SES with Uncontrolled SLE” profile (b = 0.72, SE = 0.23, 95% CI = 0.27, 1.17).

### Sensitivity analyses

Separate sensitivity analyses were conducted among survivors and the deceased. Results of proportional hazard models among survivors only (*n* = 403) were consistent with models that included deceased participants (Table S2). Poisson regression estimates among survivors were also consistent with full-sample models (Table S2), except for “Younger/Lower SES with Uncontrolled SLE” participants, whose estimate decreased in magnitude and statistical significance (b = 0.38, SE = 0.24, 95% CI = −0.09, 0.84).

Twenty-four participants died during the study period. Most (*n* = 15; 62.5%) were “Older/Lower SES with Uncontrolled SLE,” followed by eight participants (33.3%) in the “Younger/Lower SES with Uncontrolled SLE” profile. Only 1 deceased participant (4.2%) was classified as “Mid-SES with Controlled SLE” and none were “Higher SES with Controlled SLE,” thus precluding reliable regression estimates for both profiles. Results of proportional hazard and logistic regression models comparing “Older/Lower SES with Uncontrolled SLE” and “Younger/Lower SES with Uncontrolled SLE” participants indicate no difference in the timing of death (HR = 1.01; 95% CI = 0.40, 2.57), nor the likelihood of death over the study period (OR = 1.00, 95% CI = 0.41, 2.43).

We also conducted supplemental analyses to explore whether baseline differences in access to care and disease management across sociodemographic profiles accounted for associations with new organ damage. Proportional hazard and Poisson regression models that control for health insurance status and glucocorticoid, hydroxychloroquine, and immunosuppressant use, revealed estimates that were consistent with those of initial models.

## Discussion

The objectives of this study were to identify distinct sociodemographic profiles of Black/African American women with SLE and examine prospective associations with organ damage accrual over 2 years. Our sample is from the BeWELL Study—the largest study on the social epidemiology of SLE exclusively among Black/African American women. Examining within-group variation is important for identifying mechanisms that generate racial inequities in SLE progression and outcomes.^31^ We found that the timing and extent of organ damage accrual varied by sociodemographic profiles.

Older Black/African American women with fewer socioeconomic resources and uncontrolled disease activity faced the highest risk for earlier and greater organ damage accrual over time, which could not be explained by differences in medication use or health insurance. Additionally, younger, lower SES participants with uncontrolled SLE but relatively shorter disease duration also experienced elevated risk of organ damage accrual. Interventions to reduce disease activity among socioeconomically disadvantaged Black/African American women may prevent the accumulation of organ damage and reduce racial disparities in SLE outcomes. Moreover, given existing organ damage increases the risk of new organ damage, targeted efforts to mitigate disease activity specifically among younger, lower SES Black/African American women with uncontrolled SLE may effectively prevent their transition into the higher-risk, older sociodemographic profile.^8,9^ Collectively, this study adds to evidence that indicates the assessment of salient social determinants of health, including socioeconomic resources, may be useful in clinical practice for identifying subgroups at higher risk for disease progression.^32^

Our findings align with past research on socioeconomic correlates of organ damage accrual in primarily white or racially diverse SLE samples.^33^ Living in poverty has been prospectively associated with increased damage accumulation and in turn, mortality; and that exiting poverty confers less damage accrual compared to those who remain in poverty.^20,34^ Other research found that socioeconomic factors (education, household income, and health insurance) contributed to greater, earlier, and faster organ damage accrual experienced by Black/African American patients over 13 years since diagnosis, on average.^10^ Our study advances this line of research by demonstrating heterogeneity in the critical role socioeconomic resources play in organ damage accrual among Black/African American women who experience inequitable disease burden.

Socioeconomic disadvantage can hasten organ damage accrual and SLE progression through multiple pathways. SLE activity is sensitive to psychosocial stressors, including those stemming from living in or near concentrated poverty, such as perceived neighborhood disorder and exposure to crime which have been linked to adverse health outcomes for people living with SLE.^18,35^ Work disability due to SLE is also common and can have socioeconomic consequences, including lost access to employer-provided private health insurance and reduced income.^36,37^ Financial strain can result in prioritization of housing, food, and other basic necessities over medication adherence and other recommendations for optimal SLE monitoring.^35^ Medication non-adherence also has been found to be higher among Medicaid recipients, those with greater comorbid conditions and physical limitations, and those living in racially segregated neighborhoods.^38,39^ Other research has shown that many SLE patients choose to discontinue or are non-adherent to treatment regimens due to fear of adverse side effects, lack of efficacy, perceived lack of respect or compassion from providers, depression, and increasing cost and complexity of care related to longer disease duration.^40–44^ Access to care has also been shown to be lowest among older SLE patients of low socioeconomic position, and many high-risk patients often do not receive the persistent preventative care they require.^45,46^ Together, these factors may partially explain the lower medication use we observed among Older/Lower SES with Uncontrolled SLE participants.

More than two-thirds of Black/African American women in our sample were assigned to profiles characterized by lower levels of education, higher rates of work disability, no health insurance, and household income levels at or near the federal poverty threshold. We found that these participants, particularly those who were older, faced the greatest risk of organ damage accrual. In contrast, one-third of participants lived above the poverty threshold and had controlled disease activity faced lower risk of organ damage accrual. Notably, only 11% of the sample was classified as relatively affluent with controlled SLE, and the low prevalence of socioeconomically disadvantaged participants with controlled SLE precluded our ability to identify this specific subgroup. Our findings are in line with other research that indicates Black/African American women with SLE report high levels of socioeconomic disadvantage.^10,47,48^

According to ecosocial theory, Black/African American women living with SLE face greater disease burden because of underlying social inequities generated and reinforced by historical, sociopolitical, and economic factors across time, space, and levels of society.^49^ For instance, higher rates of poverty among Black/Africans Americans are directly linked to historical (e.g., redlining) and contemporary (e.g., racial discrimination in employment) forms of structural racism that reduce opportunities for upward economic mobility.^22,23^ Beyond material socioeconomic resources, living in poverty also confers exposure to additional risk factors for SLE progression, such as neighborhood stressors and reduced access to health promoting resources via racial residential segregation.^18,35^ Ecosocial theory posits that exposure to poverty and other material and psychosocial hazards become embodied over time, thus accelerating SLE progression and shaping patterns in the population-level distribution of disease burden.^49^ Accordingly, our study contributes to a growing body of research that indicates racial inequities in SLE progression and outcomes are shaped by social factors, particularly those that are generated and reinforced by racism.^14–18,50^

This study has several limitations. We are unable to infer directionality and causal relationships between disease severity and socioeconomic resources. It is possible that few socioeconomic resources exacerbate SLE activity and damage; as well as the converse, that greater SLE severity at baseline results in adverse socioeconomic consequences, such as work loss and poverty. Although analyses account for SLE disease severity at baseline, we were unable to account for the duration of uncontrolled disease prior to baseline, which can influence future organ damage and potentially confound estimates. Specific intervention targets among the high-risk groups cannot be deduced through our analysis (e.g., controlling disease activity and/or increasing access to socioeconomic resources); however, our person-centered analytic approach facilitated the identification of profiles which may be particularly useful for clinical practice. Future research on organ damage accrual should examine psychosocial risk and protective factors for adverse disease outcomes that are salient in the lives of Black/African American women, such as racial discrimination and social support.^14,19^

Organ damage is an important SLE outcome, yet few studies have considered sociodemographic predictors of racial inequities in damage accumulation, and those that do predominantly focus on clinical predictors among racially diverse samples.^12,33^ Our study advances the social epidemiology of SLE by identifying specific subgroups of Black/African American women at greatest risk of disease progression that would otherwise go undetected using between-race study designs or variable-centered approaches that aggregate scores of individual factors. Findings from this study can be used to identify Black/African American women who may benefit the most from tailored interventions which reduce disease activity and prevent new organ damage.^32^ At the structural level, policies that address underlying social inequities and facilitate upward mobility for Black/African Americans are likely to reduce racial inequities in SLE outcomes.

## Supplemental Material

Supplemental Material - Sociodemographic profiles and organ damage accural in the Black Women’s Experience Living with Lupus study Click here for additional data file. Supplemental Material for Sociodemographic profiles and organ damage in the Black women’s experience living with lupus study by Connor Martz, Tamika Webb-Detiege, Maria I Danila and David H Chae in Lupus
