# Supplementary material for: Sociodemographic profiles and organ damage accural in the Black Women’s Experience Living with Lupus study
Source: Lupus. 2023 Dec 4;33(1):17–25. doi: 10.1177/09612033231218923 (PMC10777614; doi:10.1177/09612033231218923)
Supplement: Supplemental Material - Sociodemographic profiles and organ damage accural in the Black Women’s Experience Living with Lupus study [file sj-pdf-1-lup-10.1177_09612033231218923.pdf]

Table S1. Model fit statistics for latent profile analysis of sociodemographic and socioeconomic characteristics in the BeWELL Study (n=438).

Profile Size	Profile Proportions	BIC	ABIC	Entropy	BLRT *p*
2	.71/.29	18943.68	18804.04	0.847	0.0000
3	.47/.27/.26	18802.38	18599.27	0.807	0.0000
4	.45/.24/.19/.11	18729.39	18462.81	0.835	0.0000
5*	.40/.21/.18/.12/.08	18.731.83	18401.78	0.863	0.0000

Note: * convergence issues were encountered.

Table S2. Results of proportional hazard and Poisson regression sensitivity analyses examining associations between profile membership and time to first damage accrual and cumulative damage accrual among survivors in the BeWELL Study (n=403)

Proportional Hazard Models		
Profile Comparison	Hazard Ratio	95% CI
Younger/Lower SES with Uncontrolled SLE vs Higher SES with Controlled SLE	1.36	(0.79, 2.35)
Older/Lower SES with Uncontrolled SLE vs Higher SES with Controlled SLE	2.22	(1.27, 3.89)
Mid-SES with Controlled SLE vs Higher SES with Controlled SLE	0.98	(0.52, 1.82)

Poisson Regression Models		
Profile	b (SE)	95% CI
Younger/Lower SES with Uncontrolled SLE	0.38 (0.24)	(-0.09, 0.84)
Older/Lower SES with Uncontrolled SLE	0.75 (0.24)	(0.27, 1.23)
Mid-SES with Controlled SLE	0.01 (0.27)	(-0.53,0.54)
Higher SES with Controlled SLE	(ref)	(ref)

Note: proportional hazard models examine the time to first new organ damage accrual.

Poisson regression models examine the amount of new organ damage accrual over the two-year study period.
